# Functional level at admission is a predictor of survival in older patients admitted to an acute geriatric unit

**DOI:** 10.1186/1471-2318-12-32

**Published:** 2012-06-25

**Authors:** Lars E Matzen, Ditte B Jepsen, Jesper Ryg, Tahir Masud

**Affiliations:** 1 Department of Geriatric Medicine, Odense University Hospital, DK-5000, Odense C, Denmark; 2 University of Southern Denmark, Odense, Denmark; 3 Department of Geriatric Medicine, Nottingham University Hospital, Nottingham, UK

**Keywords:** ADL, Barthel Index, Charlson Index, Co-morbidity, Elderly, Functional assessment, Geriatric, Mortality, Survival

## Abstract

**Background:**

Functional decline is associated with increased risk of mortality in geriatric patients. Assessment of activities of daily living (ADL) with the Barthel Index (BI) at admission was studied as a predictor of survival in older patients admitted to an acute geriatric unit.

**Methods:**

All first admissions of patients with age >65 years between January 1^st^ 2005 and December 31^st^ 2009 were included. Data on BI, sex, age, and discharge diagnoses were retrieved from the hospital patient administrative system, and data on survival until September 6^th^ 2010 were retrieved from the Civil Personal Registry. Co-morbidity was measured with Charlson Co-morbidity Index (CCI). Patients were followed until death or end of study.

**Results:**

5,087 patients were included, 1,852 (36.4%) men and 3,235 (63.6%) women with mean age 81.8 (6.8) and 83.9 (7.0) years respectively. The median [IQR] length of stay was 8 days, the median follow up [IQR] 1.4 [0.3; 2.8] years and in hospital mortality 8.2%. Mortality was greater in men than in women with median survival (95%-CI) 1.3 (1.2 -1.5) years and 2.2 (2.1-2.4) years respectively (p < 0.001). The median survivals (95%-CI) stratified on BI groups in men (n = 1,653) and women (n = 2,874) respectively were: BI 80-100: 2.6 (1.9-3.1) years and 4.5 (3.9-5.4) years; BI 50-79: 1.7 (1.5-2.1) years and 3.1 (2.7-3.5) years; BI 25-49: 1.5 (1.3-1.9) years and 1.9 (1.5-2.2) years and BI 0-24: 0.5 (0.3-0.7) years and 0.8 (0.6-0.9) years. In multivariate logistic regression analysis with BI 80-100 as baseline and controlling for significant covariates (sex, age, CCI, and diseases of cancer, haematology, cardiovascular, respiratory, infectious and bone and connective tissues) the odds ratios for 3 and 12 months survival (95%-CI) decreased with declining BI: BI 50-79: 0.74 (0.55-0.99) (p < 0.05) and 0,80 (0.65-0.97)(p < 0.05); BI 25-49: 0.44 (0.33-0.59)(p < 0.001) and 0.55 (0.45-0.68)(p < 0.001); and BI 0-24: 0.18 (0.14-0.24)(p < 0.001) and 0.29 (0.24-0.35)(p < 0.001) respectively.

**Conclusion:**

BI is a strong independent predictor of survival in older patients admitted to an acute geriatric unit. These data suggest that assessment of ADL may have a potential role in decision making for the clinical management of frail geriatric inpatients.

## Background

The combination of acute and chronic diseases in the ageing individual often results in disabilities and limitations in activities of daily living (ADL) [1]. Different co-morbidity indexes can measure this heterogeneity and be used in prognosis estimation [2]. However, they are time consuming in practice and difficult to implement in daily use.

Functional limitations are associated with mortality in patients with hip fractures [3], pulmonary infections [4,5] and acute medical patients [6,7]. The Barthel Index (BI) [8] is an easy to use instrument originally developed to measure ADL in stroke patients, but subsequently its use has been extended into geriatric patients [9]. In Denmark BI is the official ADL tool included in the Diagnosis Related Groups for hospitals reimbursement of geriatric patients.

The health care system in the western world will in the years to come face an increasing number of older people with chronic diseases [10], and simple and easy to use instruments to predict prognosis may be helpful in planning the optimal management of geriatric patients, both to the benefit of the individual patient but also to the benefit of health care expenses. The aim of this study was to evaluate whether the routine use of BI could be included as a prognosis indicator in terms of survival in geriatric patients.

## Methods

### Data collection

Between January 1^st^ 2005 and December 31^st^ 2009 7,723 patients were admitted to the Department of Geriatric Medicine at Odense University Hospital. Patients first admission with a length of stay > one day and age >65 years were included in this study (n = 5,087). The hospital serves a population of about 300.000 citizens. Up to June 1^st^ 2008 patients were referred directly to the geriatric wards, thereafter admitted to the acute medical ward and within 24 hours transferred to the geriatric wards, based on either daytime assessment by geriatricians or written algorithms (acute medical problems, multi co-morbidities and functional limitations).

In Denmark every citizen at birth or immigration is given a unique Civil Personal Registry Code which will identify the person in every contact with the health care system. From the hospital Patients Administrative System, data on sex, age, date of admission and diagnoses were collected. Patients were followed until death or end of study at September 6^th^ 2010. Data on survivals were retrieved from the Civil Personal Registry, and survival times from date of first admission were calculated.

Diagnoses were grouped according to the International Classification of Diseases 10^th^ revision (ICD-10) in primary diagnoses describing the leading disease during hospital stay, secondary diagnoses describing other important diseases and the combination of either primary- or secondary-diagnoses (Appendix 1).

### Co-morbidity index

Co-morbidity was measured with the Charlson Co-morbidity Index (CCI) which was calculated from ICD-10 diagnoses retrieved from the Patient Administrative System (Appendix 2) [11,12]. The validity of the calculated CCI was tested by one of the authors (DJ), who identified all existing diagnoses by reviewing a sample of 95 patients records. Using this as the references, the ICD-10 diagnoses from the Patient Administrative System identified 82% of patients with chronic pulmonary diseases, 78% with congestive heart diseases, 71% with dementia, 67% with upper gastrointestinal ulcer diseases, 62% with cerebrovascular diseases, 52% with ischaemic heart diseases, 47% with diabetes and 42% with present or former malignancies. The correlation coefficient between the review and the CCI calculated from ICD-10 diagnoses was 0.67 (Spearmann, p < 0.001).

### Activities of daily living

ADL was measured with BI [8] and scored by nurses or nursing assistants within 48 hours after admission on the basis of their observations and/or self-report from patients and/or proxies. The BI is a sum score of 10 ADL items: mobility, stair climbing, transfer, feeding, bathing, toilet use, bowel function, bladder function and grooming with a range from 0 (dependent) to 100 (independent). Individual BI had been measured in 4,527 (89%) patients and scorings were grouped in four ADL groups (ICD-10 diagnoses): “Very low ADL”: BI: 0-24 (R673), “Low ADL”: BI: 25-49 (R672), “Moderate reduced ADL”: BI: 50-79(R671) and “Independent ADL”: BI: 80-100(R670). BI is part of the Danish Diagnosis Related Group algorithm for geriatric patients including 1): Multi-morbidity defined as diseases within more than two ICD-10 main groups, 2): ADL at admission measured with BI, and 3): Interventions for rehabilitation.

### Statistical analysis

Basic data handling with creation of ICD-10 groups and CCI were performed using SPSS, version 18.0. Variables with normal distributions are reported as mean (SD). Variables not normally distributed are reported as median [percentiles 25% and 75%]. Kaplan-Meier survival analysis and logistic regression analysis using STATA version 12.0. Median survivals with 95% confidence interval (95%-CI) are estimated from Kaplan-Meier plots. In the logistic regression analysis the dependent variables were survival < = 3 months and < = 12 months from admission. Odds ratios (95%-CI) from multivariate analysis are reported. All independent variables (sex, age, CCI, ICD-10 diagnosis) were included in the analysis and in successive steps removed at p >0.05. ICD-10 diagnoses were included both as single primary- and secondary-diagnosis and as the combinations of.

The study was approved by the Danish Data Protection Agency (number: 2010-41-5195). According to the Danish Law on Medical Ethics informed consent was not needed as only existing registry data from patient records, Patient Administrative System and Civil Personal Registry were used.

## Results

5,087 patients were included, 1,852 (36.4%) men and 3,235 (63.6%) women with mean age (SD) 81.8 (6.8) and 83.9 (7.0) years respectively (Table 1). The median [IQR] length of stay was 8 [5; 13] days.

**Table 1:** Basic data on 5,087 patients admitted to a geriatric department

	**Male (n = 1,852)**	**Female (n = 3,235)**	
**Age**, mean (SD)	81.8 (6.8)	83.9 (7.0)	< 0.001
**Length of stay**, median [IQR]	8 [5; 14]	8 [5; 13]	n.s.
**Barthel-Index**			
80-100	296 (17.9%)	523 (18.2%)	n.s.
50-79	452 (27.3%)	856 (29.8%)	
25-49	363 (22.0%)	643 (22.4%)	
0-24	542 (32.8%)	852 (29.7%)	
**Survival status**			
Alive 6^th^-september 2010	563 (30.4%)	1,322 (40.9%)	< 0.001
Discharged and dead before 6^th^ sept. 2010	1,115 (60.2%)	1,670 (51.6%)	
Dead in hospital	174 (9.4%)	243 (7.5%)	
**Charlson Comorbidity Index**			
0	443 (23.9%)	1,025 (31.7%)	<0.001
1	617 (33.3%)	1,205 (37.3%)	
2	429 (23.2%)	621 (19.2%)	
> = 3	363 (19.6%)	384 (11.9%)	
**ICD-diagnosis group**			
Infectious diseases (A00 – B99)	134 (7.2%)	256 (7.9%)	n.s.
Any cancer (C00 – C75)	372 (20.1%)	485 (15.0%)	< 0.001
Haematological disease (D50 – D89)	322 (17.4%)	465 (14.4%)	< 0.01
Endocrine diseases (E00 – E99)	636 (34.3%)	1,317 (40.7%)	< 0.001
Psychiatric diseases (F00 – F99)	488 (26.4%)	822 (25.4%)	n.s.
Neurologic diseases (G00 – G99)	160 (8.6%)	204 (6.3%)	< 0.01
Cardiovascular diseases (I00 – I99)	1,242 (67.1%)	2,066 (63.9%)	< 0.05
Respiratory diseases (J00 – J99)	738 (39.8%)	1,007 (31.1%)	< 0.001
Gastrointestinal diseases (K00 – K93)	291 (15.7%)	527 (16.3%)	n.s.
Bone and Connective tissue (M00 – M99)	271 (14.6%)	912 (28.2%)	< 0.001
Urologic diseases (N00 – N99)	532 (28.7%)	682 (21.1%)	< 0.001
**Number of ICD-diagnosis (mean, SD)**	4.4 (1.4)	4.3 (1.4)	n.s.

Follow up after first admission was median [IQR] 1.4 [0.3; 2.8] years corresponding to 8,960 patients years. Overall mortality in hospital was 8.2% (417/5,087) and during follow up 54,8% (2,785/5,087) (Table 1). Except for Diseases of Bone and Connective Tissue most diseases were more common in men, who had a CCI above 2 in 42.8% of men as compared to 31.1% of women (Table 1).

In 560 patients (199 men and 361 women) BI had not been measured. These patients did not differ according to sex, age, CCI or length of stay from the 4,527 (89%) patients [1,653 (36.5%) men and 2,874 (63,5%) women] included in the survival and logistic regression analyses.

**Figure 1:**
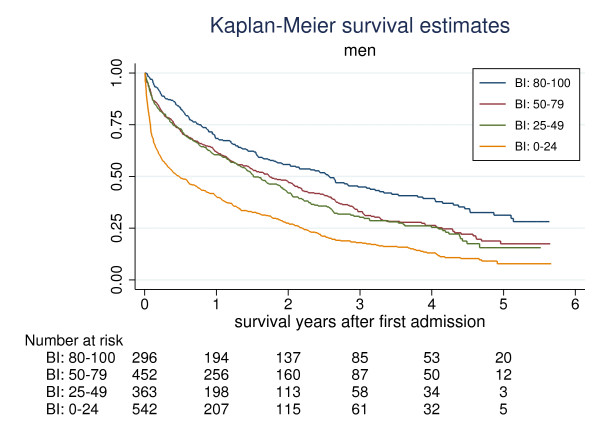
**Kaplan-Meier survival estimates in men stratified on Barthel Index (BI) measured at admission.** BI: 80-100 (blue), 50-79 (red), 25-49 (green), 0-24 (yellow)

**Figure 2:**
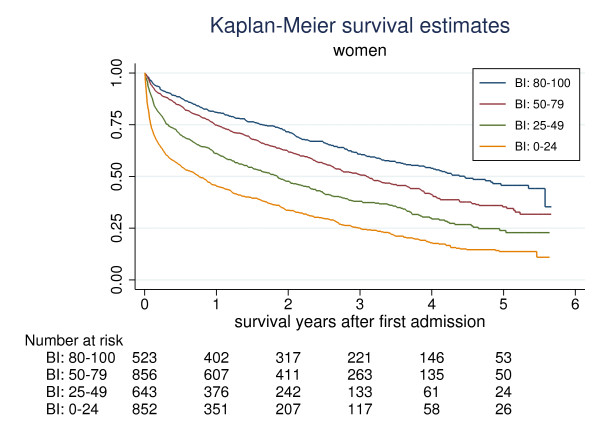
**Kaplan-Meier survival estimates in women stratified on Barthel Index (BI) measured at admission.** BI: 80-100 (blue), 50-79 (red), 25-49 (green), 0-24 (yellow)

Mortality was greater in men than in women with survival median (95%-CI) 1.3 (1.2-1.5) years and 2.2 (2.1-2.4) years respectively (Kaplan-Meier, p < 0.001). In both sexes survival were associated with BI (Figures 1 and 2). The median (95%-CI) survivals stratified on BI groups in men and women respectively were: BI 80-100: 2.6 (1.9-3.1) years and 4.5 (3.9-5.4) years; BI 50-79: 1.7 (1.5-2.1) years and 3.1 (2.7-3.5) years; BI 25-49: 1.5 (1.3-1.9) years and 1.9 (1.5-2.2) years and BI 0-24: 0.5 (0.3-0.7) years and 0.8 (0.6-0.9) years.

Survival 3 and 12 months after admission decreased with decreasing BI. With BI 80-100 as baseline the unadjusted OR (95%-CI) of surviving 3 and 12 month decreased from BI 50-79: 0.65 (0.49 – 0.86) (p < 0.01) and 0.73 (0.60 – 0.88) (p < 0.01) respectively, BI 25-49: 0.40 (0.30 – 0.53) (p < 0.001) and 0.49 (0.40 0.60) (p < 0.001) respectively to BI 0-24: 0.16 (0.12 0.21) (p < 0.001) and 0.25 (0.21 0.30) (p < 0.001) respectively. In the multivariate logistic regression analysis the association between decreasing OR´s of survival and decreasing BI were still present (Table 2). Other independent variables decreasing 3 month survivals were increasing age, increasing CCI, diagnoses of cancer, cardiovascular and respiratory diseases. Independent variables decreasing 12 months survival were male sex, diagnoses of cancer, diagnoses of haematology and respiratory diseases (Table 2). In this group of patients infectious diseases and diseases of bone and connective tissues were associated with increased 3 and 12 month survivals (Table 2).

**Table 2:** Multivariate logistic regression analysis: Odds Ratio (OR) and 95% confidence interval (95%-CI) of 3 and 12 month survival after date of admission in geriatric patients (n = 4,527)

		**Survival 3 month**		**Survival 12 month**	
		**OR**	**95%-CI**	**OR**	**95%-CI**
Barthel Index	Baseline: 80-100				
	50-79	0.74	(0.55 – 0.99)*	0.80	(0.65 – 0.97)*
	25-49	0.44	(0.33 – 0.59)***	0.55	(0.45 – 0.68)***
	0-24	0.18	(0.14 – 0.24)***	0.29	(0.24 – 0.35)***
Sex	Male (0), Female (1)	1.11	(0.95 – 1.31) n.s.	1.27	(1.11– 1.45)***
Age	years	0.96	(0.95 – 0.97)***	0.97	(0.96 – 0.98)***
Charlson Comorbidity Index	Baseline: 0				
	1	0.96	(0.78 – 1.19) n.s.	0.83	(0.71 – 0.98)*
	2	0.62	(0.48 – 0.78)***	0.63	(0.52 – 0.77)***
	> = 3	0.53	(0.40 – 0.70)***	0.51	(0.40 – 0.64)***
ICD-10 diagnoses					
Cancer	Diagnoses (C00-D48)	0.33	(0.25 – 0.42)***	0.38	(0.29 – 0.49)***
Cardiovascular diseases	A-diagnoses (I00-I99)	0.62	(0.50 – 0.77)***		
Haematology	Diagnoses (D50-D89)			0.77	(0.65 – 0.92)**
Respiratory Diseases	Diagnoses (J00-J99)	0.59	(0.50 – 0.69)***	0.67	(0.59 – 0.77)***
Infectious Diseases	Diagnoses (A00-B99)	2.59	(1.85 – 3.64)***	2.24	(1.78 – 3.36)***
Bone and Connective Tissue Diseases	Diagnoses (M00-M79, M86-M99)	1.54	(1.26– 1.87)***	1.24	(1.07 – 1.45)**

***: p < 0.001, **: p < 0.01, * p < 0.05.

A-diagnosis: Primary ICD-10 discharge diagnosis.

B-diagnosis: Secondary ICD-10 discharge diagnosis.

Independent variables: activities of daily living (ADL-group) measured with Barthel-Index, sex, age, comorbidity measured by Charlson Comorbidity Index and ICD-10 diagnoses.

## Discussion

The main finding in this study was the strong and independent association between ADL function and survival in geriatric patients. The BI could differentiate between groups with a good or bad prognosis in terms of survival. The probability of being alive 3 month after admission was 75% with BI 50-79, 44% with BI 26-49 and only 18% with BI 0-25. The corresponding figures for being alive 12 month after admission were 80%, 54% and 28%. However, as shown in Figures 1 and 2 the prognosis in an individual patient would still be difficult to estimate and even in patients with BI below 25, more than 25% of male and female patients were alive after 2.2 and 3.0 years respectively. As shown in the Kaplan-Meier graphs, the four BI groups discriminated better in women than in men. In the latter, the survivals of men in the BI 50-79 and BI 25-49 were the same.

The patients in this study were a selected cohort of older medical patients. However, they were representative of a typical case-mix population admitted to an acute geriatric department [13-17]. The strength of this study was the inclusion of 4,527 patients in the survival analysis and the ability to collect data from the Danish national databases of diagnoses and mortality. A limitation is that the data were generated in the clinical setting with associated reporting uncertainties in diagnoses.

The associations between functional status or frailty, multi-morbidity and mortality have been described previously in study settings [4-6,18,19]. Walter et al. [20] found that functional status at discharge, male sex, cancer, hearth diseases, creatinine and albumin at admission were predictive of one year survival. In a prospective study of 463 acute medical patients with a median CCI of 2.1, Buurman at al. [6] found that the risk of mortality was associated with four clinical variables – functional level measured with BI, co-morbidity, malignancy and serum urea nitrogen level. Raineri et al [4] found in a prospective study of 244 patients with mean age 81.7 years and acute exacerbations in COPD, that risk factors for 6 month mortality were BMI, BI at discharge and morbidity measured with Apache II. Torres et al. [5] found in elderly patients with pneumonia, that high BI was related to reduced 30-day and 18 month mortalities. Our results were consistent with these studies showing the same associations between mortality and functional status, co-morbidity and single diseases of cancer, cardiovascular, respiratory and haematology. The positive odds ratios of infectious diseases and diseases of bone and connective tissues on both 3 and 12 month survivals indicated that the former normally were cured and the latter might be troublesome but not lethal.

Male sex, age and co-morbidity measured with CCI were associated, independently of functional status, with decreased survival and the survival odds ratios decreased with increasing CCI. The association between co-morbidity and survival was also found previously in a study on 1,567,659 admissions [21], but in contrast to our study data on functional status was lacking.

BI scorings were performed by nurses or nursing assistants and the methods used were observations of patient performances, interview with patients and/or proxies. BI data had been reported in 89% of our patients and as part of the DRG reimbursement the nursing staff were instructed to accurately score the BI, when receiving new patients in the wards. All new staff members were instructed in BI scoring at employment. Although we had no data on the reliability of this method in our department, others have found acceptable interrater reliability of BI scoring done by nonclinical assistants [22] and when scoring was performed by different observers as in our department [9].

In this study we had data only on the four ADL diagnoses groups and not on the ten BI sub-items. Therefore, it was not possible to ascertain whether some of the items had greater impact than the others. The study was based on data obtained in clinical setting, and imprecision in the BI sum score might influence the allocation to ADL diagnoses groups, but probably not the strong association between BI and survival.

In a study using ICD-10 diagnoses from the Patient Administrative System some uncertainties in diagnosis reporting are inevitable. The meticulous review of 95 patient records gave a higher CCI than the one created from ICD-10 diagnoses. However, the coefficient of correlation was 0.67 and ICD-10 calculated CCI was accepted as a proxy for co-morbidity. The validity of primary diagnosis in the Danish Registry of Discharge Diagnosis has been found to be high [12].

## Conclusion

Functional status at admission in combination with demographic factors and comorbidities could potentially be used in the decision making process on further management of geriatric patients. Acute medical conditions should of course be treated without hesitation. However knowledge of functional status and its predicting ability in terms of survival may influence decisions on factors such as the extent of invasive investigations, treatments associated with significant risk, and adding to the poly-pharmacy burden of elderly patients. These issues require further exploration.

## Competing interest

Lars Erik Matzen: None

Ditte Beck Jepsen: None

Jesper Ryg: None

Tahir Masud: None

## Authors’ contributions

LEM, Head of Department, Associate Professor: Conception and design, acquisition of data, data analysis and interpretation of data, drafting manuscript, critical revising manuscript, final approval. DBJ, medical student: Validation of diagnosis and Charlson Index, final approval. JR, Post-Doc, Ph.D.: Conception and design, interpretation of data, critical revising manuscript for intellectual content, final approval. TM, Professor, Ph.D.: Conception and design, interpretation of data, critical revising manuscript for intellectual content, final approval. All authors read and approved the final manuscript.

## Appendix 1

Grouping of ICD-10 diagnosis: Infectious Diseases (A00-B99), Cancer (C00-D48), Haematology (D50-D89), Endocrine (E00-E99), Psychiatric including Dementia (F00-F99, G300-G309), Neurology (G00-G30, G31-G99), Cardiovascular Diseases (I00-I99), Respiratory Diseases (J00-J99), Gastrointestinal Diseases (K00-K93), Dermatology (L00-L99), Bone and Connective Tissue Diseases (M00-M79, M86-M99) and Urology (N00-N99).

### Appendix 2

Charlson Index (CCI) calculated from Patient Administrative System ICD-10 diagnoses (rank; ICD-10 number): Ischaemic Heart Diseases and Myocardial Infarction (1; I21, I22, I23, I25), Congestive Heart Failure (1; I50, I110, I130 I132), Peripheral Vascular Diseases (1; I70, I71, I72, I73, I74, I77), Cerebrovascular Diseases (1; I60-I69, G45, G46), Dementia (1; F00-F03, F051, G30), Chronic Pulmonary Diseases (1; J40-J47, J60-J67, J684, J70.1, J703, J84.1, J920, J961, J982, J983), Diabetes uncomplicated (1; E10, E101, E109, E110, E111, E119, E140, E141, E149), Connective Tissue Diseases (1; M05, M06, M08, M09, M30-M36, D86), Upper Gastro-intestinal and Ulcer Diseases (1; K221, K25-K28), Mild Liver Diseases (1; B18, K70-K703, K709, K71, K73, K74, K76), Hemiplegia (2; G81, G82), Moderate/severe Renal Diseases (2; I12, I13, N00-N05, N07, N11, N14, N17-N19, Q61), Diabetes Mellitus with Chronic Complications (2; E102-E108, E112-E118, E142-E148), Leucaemia (2; C91-C95), Lymphoma (2; C81-C85, C88, C90, C96), Moderate/severe Liver Diseases (2; B150, B160, B162, B190, K704, K72, K766, I85), Metastatic solid Cancer (6; C76-C80) and AIDS (6; B21-B24).

## Pre-publication history

The pre-publication history for this paper can be accessed here:

http://www.biomedcentral.com/1471-2318/12/32/prepub

## References

[B1] Verbrugge LM Jette AM The disablement process Soc Sci Med 1994 38 1 14 10.1016/0277-9536(94)90294-1 8146699

[B2] Zekry D Loures Valle BH Lardi C Graf C Michel JP Gold G Krause KH Herrmann FR Geriatrics index of comorbidity was the most accurate predictor of death in geriatric hospital among six comorbidity scores J Clin Epidemiol 2010 63 1036 1044 10.1016/j.jclinepi.2009.11.013 20236800

[B3] Formiga F Chivite D Sole A Manito N Ramon JM Pujol R Functional outcomes of elderly patients after the first hospital admission for decompensated heart failure (HF). A prospective study Arch Gerontol Geriatr 2006 43 175 185 10.1016/j.archger.2005.10.010 16356561

[B4] Ranieri P Bianchetti A Margiotta A Virgillo A Clini EM Trabucchi M Predictors of 6-month mortality in elderly patients with mild chronic obstructive pulmonary disease discharged from a medical ward after acute nonacidotic exacerbation J Am Geriatr Soc 2008 56 909 913 10.1111/j.1532-5415.2008.01683.x 18384582

[B5] Torres OH Munoz J Ruiz D Ris J Gich I Coma E Gurgui M Vazquez G Outcome predictors of pneumonia in elderly patients: importance of functional assessment J Am Geriatr Soc 2004 52 1603 1609 10.1111/j.1532-5415.2004.52492.x 15450034

[B6] Buurman BM van Munster BC Korevaar JC Abu-Hanna A Levi M de Rooij SE Prognostication in acutely admitted older patients by nurses and physicians J Gen Intern Med 2008 23 1883 1889 10.1007/s11606-008-0741-7 18769983 PMC2585689

[B7] Espaulella J Arnau A Cubi D Amblas J Yanez A Time-dependent prognostic factors of 6-month mortality in frail elderly patients admitted to post-acute care Age Ageing 2007 36 407 413 10.1093/ageing/afm033 17395620

[B8] Mahoney FI Barthel DW Functional Evaluation: The Barhel Index Md State Med J 1965 14 61 65 61-65 14258950

[B9] Sainsbury A Seebass G Bansal A Young JB Reliability of the Barthel Index when used with older people Age Ageing 2005 34 228 232 10.1093/ageing/afi063 15863408

[B10] Christensen K Doblhammer G Rau R Vaupel JW Ageing populations: the challenges ahead Lancet 2009 374 1196 1208 10.1016/S0140-6736(09)61460-4 19801098 PMC2810516

[B11] Charlson ME Pompei P Ales KL MacKenzie CR A new method of classifying prognostic comorbidity in longitudinal studies: Development and validation J Chron Dis 1987 40 373 383 10.1016/0021-9681(87)90171-8 3558716

[B12] Thygesen SK Christiansen CF Christensen S Lash TL Sorensen HT The predictive value of ICD-10 diagnostic coding used to assess Charlson comorbidity index conditions in the population-based Danish National Registry of Patients BMC Med Res Methodol 2011 11 83 83 10.1186/1471-2288-11-83 21619668 PMC3125388

[B13] Saltvedt I Saltnes T Mo ES Fayers P Kaasa S Sletvold O Acute geriatric intervention increases the number of patients able to live at home. A prospective randomized study Aging Clin Exp Res 2004 16 300 306 15575124 10.1007/BF03324555

[B14] Baztan JJ Suarez-Garcia FM Lopez-Arrieta J Rodriguez-Manas L Rodriguez-Artalejo F Effectiveness of acute geriatric units on functional decline, living at home, and case fatality among older patients admitted to hospital for acute medical disorders: meta-analysis BMJ 2009 338 b50 10.1136/bmj.b50 19164393 PMC2769066

[B15] Asplund K Gustafson Y Jacobsson C Bucht G Wahlin A Peterson J Blom JO Angquist KA Geriatric-based versus general wards for older acute medical patients: a randomized comparison of outcomes and use of resources J Am Geriatr Soc 2000 48 1381 1388 11083312 10.1111/j.1532-5415.2000.tb02626.x

[B16] Counsell SR Holder CM Liebenauer LL Palmer RM Fortinsky RH Kresevic DM Quinn LM Allen KR Covinsky KE Landefeld CS Effects of a multicomponent intervention on functional outcomes and process of care in hospitalized older patients: a randomized controlled trial of Acute Care for Elders (ACE) in a community hospital J Am Geriatr Soc 2000 48 1572 1581 11129745 10.1111/j.1532-5415.2000.tb03866.x

[B17] Landefeld CS Palmer RM Kresevic DM Fortinsky RH Kowal J A randomized trial of care in a hospital medical unit especially designed to improve the functional outcomes of acutely ill older patients N Engl J Med 1995 332 1338 1344 10.1056/NEJM199505183322006 7715644

[B18] Salvi F Miller MD Grilli A Giorgi R Towers AL Morichi V Spazzafumo L Mancinelli L Espinosa E Rappelli A A Manual of Guidelines to Score the Modified Cumulative Illness Rating Scale and Its Validation in Acute Hospitalized Elderly Patients J Am Geriatr Soc 2008 56 1926 1931 10.1111/j.1532-5415.2008.01935.x 18811613

[B19] Zekry D Valle BH Michel JP Esposito F Gold G Krause KH Herrmann FR Prospective comparison of six co-morbidity indices as predictors of 5 years post hospital discharge survival in the elderly Rejuvenation Res 2010 13 675 682 10.1089/rej.2010.1037 20818930

[B20] Walter LC Brand RJ Counsell SR Palmer RM Landefeld CS Fortinsky RH Covinsky KE Development and Validation of a Prognostic Index for 1-Year Mortality in Older Adults After Hospitalization JAMA 2001 285 2987 2994 10.1001/jama.285.23.2987 11410097

[B21] Barba R Martinez JM Zapatero A Plaza S Losa JE Canora J Perez A de Garcia CG Mortality and complications in very old patients (90+) admitted to departments of internal medicine in Spain Eur J Intern Med 2011 22 49 52 10.1016/j.ejim.2010.11.001 21238893

[B22] Richards SH Peters TJ Coast J Gunnell DJ Darlow MA Pounsford J Inter-rater reliability of the Barthel ADL index: how does a researcher compare to a nurse? Clin Rehabil 2000 14 72 78 10.1191/026921500667059345 10688347

